# Rotational Thromboelastometry (ROTEM^®^) in Relation to Inflammatory Biomarkers and Clinical Outcome in COVID-19 Patients

**DOI:** 10.3390/jcm12123919

**Published:** 2023-06-08

**Authors:** Pawel Rogalski, Magdalena Rogalska, Diana Martonik, Malgorzata Rusak, Joanna Pawlus, Joanna Chociej-Stypulkowska, Milena Dabrowska, Robert Flisiak

**Affiliations:** 1 Department of Gastroenterology and Internal Medicine, Medical University of Bialystok, 15-276 Białystok, Poland; 2 Department of Infectious Diseases and Hepatology, Medical University of Bialystok, 15-540 Białystok, Poland; pmagdar@gmail.com (M.R.); di.martonik@gmail.com (D.M.); robert.flisiak1@gmail.com (R.F.); 3 Department of Haematological Diagnostics, Medical University of Bialystok, 15-276 Białystok, Poland; malgorzata.rusak@umb.edu.pl (M.R.); joanna.pawlus@umb.edu.pl (J.P.); joannacs@o2.pl (J.C.-S.); mdabrows@umwb.edu.pl (M.D.)

**Keywords:** coronavirus disease-19, inflammatory biomarkers, interleukins, hypercoagulability, rotational thromboelastometry, ROTEM

## Abstract

*Background:* The pathogenesis of hypercoagulability in COVID-19 patients is complex and not fully understood. Rotational thromboelastometry (ROTEM^®^) is a viscoelastic method that allows the definition of a patient’s hemostatic profile. This study aimed to assess the relationship between ROTEM^®^ parameters, the profile of inflammatory cytokines, and clinical outcomes in COVID-19 patients. *Methods:* A total of 63 participants (*n* = 29 symptomatic non-ICU COVID-19 patients, and *n* = 34 healthy controls) were prospectively included in the study. We assessed the relationship between the parameters of three ROTEM^®^ tests (NATEM^®^, EXTEM^®^, and FIBTEM^®^) and levels of CRP, interleukin-8, interleukin-1β, interleukin-6, interleukin-10, tumor necrosis factor, interleukin 12p70, and clinical outcomes. *Results:* ROTEM^®^ indicated hypercoagulability in COVID-19 patients in all the tests performed. The levels of all inflammatory cytokines were significantly higher in COVID-19 patients. NATEM more frequently detected hypercoagulability in COVID-19 patients compared to EXTEM. The strongest correlations with inflammatory biomarkers and CT severity score were with FIBTEM parameters. The elevated maximum clot elasticity (MCE) in FIBTEM was the strongest predictor of poor outcomes. *Conclusions:* Increased FIBTEM MCE may be associated with greater severity of COVID-19. Non-activated ROTEM (NATEM test) seems to be more valuable for detecting hypercoagulability in COVID-19 patients compared to the tissue factor activated test (EXTEM).

## 1. Introduction

Coronavirus disease 2019 (COVID-19) can affect multiple systems, with respiratory failure being the most common cause of fatal outcomes. Thrombotic and thromboembolic complications are relatively common in COVID-19 patients, and their incidence increases with the severity of the disease. In the meta-analysis of 49 studies, including 18,093 patients, the reported incidence of venous thromboembolism (VTE) was 33.0% in studies with patients systematically screened for VTE [1]. The incidence of VTE in COVID-19 patients in intensive care units (ICUs) may even be higher, and it was 36–46%, according to the latest systematic reviews [2,3].

The prothrombotic profile of COVID-19 patients is associated with several factors, including excessive inflammation, endothelial cell injury, hypercoagulability, and platelet activation and impaired fibrinolysis [4]. The classic features of COVID-19-associated hemostatic abnormalities (CAHAs) include normal or slightly prolonged prothrombin time (PT) and activated partial thromboplastin time (APTT), elevated levels of fibrinogen and D-dimer; the platelet count is usually normal or elevated [5]. Markedly elevated levels of von Willebrand factor (vWf) and factor VIII are common in COVID-19 patients. Natural anticoagulants are usually slightly disturbed (small decreases in antithrombin and free protein S and a small increase in protein C levels). Although the coagulation profile of COVID patients may resemble other systemic coagulopathies, such as disseminated intravascular coagulation (DIC), sepsis-induced coagulopathy (SIC), and thrombotic microangiopathy, CAHAs have some unique features. The issue is further complicated by the fact that CAHAs can vary depending on the severity and stage of COVID-19. Moreover, local thromboembolic complications and DIC and SIC may occur as late complications of COVID-19, interfering with CAHA. Overall, the pathogenesis of hypercoagulability in COVID-19 patients is multifactorial and not fully understood.

C-reactive protein (CRP) and interleukin-6 (Il-6) levels correlate positively with COVID-19 severity and mortality. The levels of both biomarkers at admission can predict progression to critical illness [6,7]. It has also been shown that the CRP level may be valuable in predicting the risk of thrombosis [6]. However, the relationship between the level of Il-6 and other interleukins and the risk of thrombosis in COVID-19 patients has not been confirmed. 

Most basic blood clotting tests do not detect prothrombotic disorders reliably. Rotational thromboelastometry (ROTEM^®^) is a viscoelastic method that allows us to assess the quality of clots and the dynamics of clot formation from the initiation of coagulation to fibrinolysis [8]. ROTEM is a point-of-care method that allows the rapid detection of hypercoagulability, coagulation factor deficiencies, and fibrinolysis disorders [9]. In addition, using specific activators and inhibitors, ROTEM can detect fibrinogen deficiencies and abnormalities in fibrin polymerization and platelet involvement in clot formation. Previous studies have demonstrated the usefulness of viscoelastic tests for detecting hypercoagulability in patients with COVID-19. Hypercoagulability in these studies was manifested by a shortened initiation time of clot formation, more rapid clot formation, increased clot amplitude, and maximum clot firmness, and increased clot stability (prolonged fibrinolysis) in patients with COVID-19 [10]. Importantly, hypercoagulability was often detected despite the widespread use of anticoagulants [11,12,13]. Some parameters of viscoelastic tests were also found to be more useful compared to standard biomarkers in discriminating between patients with COVID-19 from patients with pneumonia without COVID-19 [14]. Nevertheless, these studies mainly focused on ICU patients. Thromboelastometry is one of the few tests that can potentially assess thromboembolic risk and patient prognosis in COVID-19 patients. Unlike most previous studies, this study focuses on non-ICU COVID-19 patients. Therefore, the results of our study can be generalized to the larger population. 

Taking into account the relationship between the severity of COVID-19 and the increased risk of thromboembolic events and the relationship between the level of inflammatory biomarkers and the severity of COVID-19, we analyzed the relationship between the level of pro-inflammatory biomarkers and ROTEM^®^ as a global test assessing many aspects of blood coagulation and fibrinolysis. 

The aim of the study was to assess the relationship between the parameters of three ROTEM^®^ tests (NATEM^®^, EXTEM^®^, and FIBTEM^®^) and the profile of inflammatory cytokines including CRP, interleukin-8 (IL-8), interleukin-1β (IL-1β), interleukin-6 (IL-6), interleukin-10 (IL-10), tumor necrosis factor (TNF), and interleukin 12p70 (IL-12p70) in non-ICU COVID-19 patients. In addition, we analyzed the relationship between the above parameters and the clinical course of COVID-19.

## 2. Materials and Methods

A total of 63 participants (*n* = 29 COVID-19 patients, and *n* = 34 healthy controls; mean age 63.24 ± 19 years; 35 (55.56%) female) were prospectively included in the study from October 2021 to January 2023. COVID-19 patients were included in the study from October 2021 to January 2022, during the period of dominant occurrence of the Delta SARS-CoV-2 variant in our country. Healthy controls were recruited from medical staff and had no infectious symptoms in the last six months prior to blood sampling. The control group was sex-matched with the COVID-19 group (*p* = 0.31). The controls were slightly younger than the COVID-19 group (55 (44; 61) vs. 66 (43; 77), *p* = 0.01).

We only included adults with symptomatic SARS-CoV2 infection confirmed by an antigen test or RT-PCR with a nasopharyngeal swab sample. All patients required hospitalization due to symptomatic COVID-19, and all were admitted to the hospital during the first seven days from the beginning of symptoms. Twenty-three (79.31%) patients had SpO2 below 95%, and eleven (40.74%) patients had a CT disease severity score >50% (at least 13/25 points). Patients were diagnosed and treated according to the applicable national recommendations for managing COVID-19 [15]. Low-molecular-weight heparin (LMWH) in prophylactic or therapeutic doses and remdesivir were administered to all (100%) patients, whereas tocilizumab was given to 9 (31.03%) patients. The exclusion criteria were as follows: (1) asymptomatic or mild COVID-19 not requiring hospitalization, (2) severe COVID-19 requiring mechanical ventilation and/or treatment in an intensive care unit, and (3) the use of anticoagulants other than low-molecular-weight heparin (e.g., chronic treatment with warfarin or direct oral anticoagulants for atrial fibrillation). 

The study was approved by the bioethics committee of our institution. All patients gave informed written consent for blood collection and analysis of the clinical data records. The study was conducted in accordance with The World Medical Association Declaration of Helsinki.

### 2.1. Blood Collection and Storage

Blood was collected by atraumatic venipuncture into vacuum tubes. Blood for ROTEM was collected in S-Monovette^®^ Citrate 9NC tubes (SARSTEDT AG&Co., Nümbrecht, Germany). Thromboelastometry was performed within 1 h of blood collection. Blood for flow cytometry was collected in S-Monovette^®^ EDTA tubes (SARSTEDT AG&Co., Nümbrecht, Germany). Immediately after collection, the blood was centrifuged for 5 min at 200 g, and the plasma was collected. The plasma samples were frozen within 30 min of centrifugation at −20 °C and stored until being assayed.

### 2.2. Rotational Thromboelastometry (ROTEM^®^)

The methodology of rotational thromboelastometry (ROTEM^®^, TEM International GmbH, Munich, Germany) has previously been described in detail. Thromboelastometry was performed according to the manufacturer’s recommendations using a ROTEM Gamma^®^ Analyzer (TEM International GmbH, Munich, Germany). All patients’ whole blood samples were analyzed by NATEM^®^, EXTEM^®^, and FIBTEM^®^ assays. NATEM^®^ is a non-activated test where clotting is initiated by contact of blood with the surface of the cuvette; the activation of coagulation in this assay is the slowest, but it allows for the detection of subtle coagulation disorders. EXTEM^®^ is a test for the analysis of blood coagulation after the activation of coagulation by tissue factor. FIBTEM^®^ is an EXTEM^®^-based assay with the addition of a platelet inhibitor (cytochalasin) used to analyze coagulation without platelets. Prior to analysis, citrated blood was stored at room temperature. Before the test, blood and cuvettes (Cup and pin cells, TEM International GmbH, Munich, Germany) were incubated at the temperature of 37 °C. All pipetting steps and the blood and reagent mixing were performed in a standardized manner by following an automated program. The sample tubes were gently inverted at least 5 times before pipetting the blood. Tests were run for 60 min. We recorded the following parameters: clotting time (CT), clot formation time (CFT), alpha angle, clot formation rate (CFR), maximum clot firmness (MCF), maximal lysis (ML) for NATEM and EXTEM tests and MCF, MCE, amplitude in 10 min (A10) and A20 for FIBTEM tests.

### 2.3. Inflammatory Cytokines—Flow Cytometric Analysis

The BD™ CBA Human Inflammatory Cytokines Kit (BD Bioscience) was used, allowing the measurement of interleukin-8 (IL-8), interleukin-1β (IL-1β), interleukin-6 (IL-6), interleukin-10 (IL-10), tumor necrosis factor (TNF), and interleukin12p70 (IL-12p70) levels. Tests were performed according to the manufacturer’s protocols. Human inflammatory cytokine kit: 50 μL of test beads, 50 μL of test sample or standard, and 50 μL of PE-labeled antibodies (detection reagent) were added sequentially to each sample tube. The samples were incubated at room temperature in the dark for 1.5 h. The samples were then washed with 1 mL of wash buffer and centrifuged, and the resulting pellet was resuspended in 50 μL of detection reagent. The samples were incubated for 1.5 h, washed again, and centrifuged. After discarding the supernatant, the pellet was resuspended in 300 μL of wash buffer and analyzed the same day in a flow cytometer (Canto II).

### 2.4. Statistical Analysis

Continuous variables were expressed as means ± standard deviation (SD) or median (interquartile range (IQR)), as appropriate. Categorical variables were reported as counts (percentage). Continuous data were analyzed with *t*-tests or Mann–Whitney U tests, as appropriate. The categorical variables were analyzed using an χ^2^ test. *p*-values of <0.05 were considered statistically significant. Spearman’s rank correlation was used to assess the correlation between the ROTEM parameters and inflammatory biomarkers and the CT severity score; the rank correlation coefficient (R) and correlation significance (p) are presented. The additional analysis included stepwise multivariate logistic regression, which was used to identify the best combination of parameters predicting death or transfer to ICU/mechanical ventilation in COVID-19 patients. The model was assessed with the χ^2^ test, R2 Nagelkerky coefficient, and Hosmer–Lemeshow goodness of fit (GOF) test. The receiver operating characteristic (ROC) curves were prepared. The cutoff point calculation was based on Youden’s criterion. Statistical analysis was performed using TIBCO Software Inc. (2017). Statistica (data analysis software system), version 13. http://statistica.io (accessed on 1 January 2023). 

## 3. Results

All patients required hospitalization for moderate to severe disease but did not require intensive care at the time of admission. Only three (10.35%) patients in the COVID-19 group were fully vaccinated. The median time from symptom onset to hospital admission was 5 (4; 6) days. The median hemoglobin saturation at admission was 92% (86; 94). Chest computed tomography was performed in all patients. The median CT disease severity score was 10 (3; 18) out of 25 points. One patient (3.45%) had a confirmed thromboembolic event (pulmonary embolism). Four (13.79%) patients required transfer to ICU and/or mechanical ventilation. Four (13.79%) patients died during hospitalization. The median hospital stay was 10 (8; 18) days. Oxygen therapy was used in all patients with COVID-19 (*n* = 20 (68.97%) passive oxygen therapy and *n* = 9 (31.03%) passive oxygen therapy followed by high-flow oxygen therapy). 

The results of the basic laboratory tests of the patients are presented in Table 1.

### 3.1. ROTEM^®^

The ROTEM results are presented in Table 2. We showed statistically significant differences in all analyzed parameters except for ML in the NATEM test. All parameters that were statistically different between the groups indicated hypercoagulability in the COVID-19 group compared to healthy controls. Both parameters of the dynamics of clot formation (shorter CT and CFT, higher alpha and CFR) and parameters of clot quality (higher MCF and MCE) indicated hypercoagulability in the COVID-19 group. In the EXTEM test, only the alpha and CFR values differed significantly, indicating a faster clotting rate in the COVID-19 group. The other parameters (CT, CFT, MCF, ML, and MCE) did not differ significantly between the groups. In the FIBTEM test (generally EXTEM with a platelet inhibitor—cytochalasin D), we showed higher values of all analyzed parameters (MCF, MCE, A10, and A20), indicating hypercoagulability in the COVID-19 group compared to healthy controls. The proportion of platelets in the clot calculated according to the formula: EXTEM MCE–FIBTEM MCE did not differ significantly between the groups: 150.5 (140; 166) vs. 148 (138; 174), *p* = 0.90. 

### 3.2. Interleukins

The levels of all analyzed interleukins, including TNF, IL-6, IL-1B, IL-8, IL-10, and IL-12p70, were significantly higher in the COVID-19 group compared to the control group (Table 3).

### 3.3. Correlations between ROTEM Parameters and CT Severity Index

Of the ROTEM parameters, we found correlations between all FIBTEM parameters and the CT severity score (see Table 4). Other ROTEM parameters did not correlate with the CT severity score.

### 3.4. Correlations between ROTEM Parameters and Levels of Inflammatory Biomarkers

**Figure 1:**
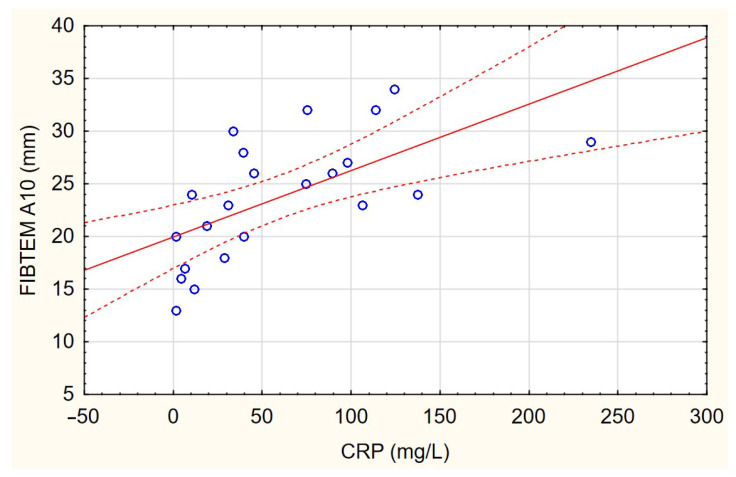

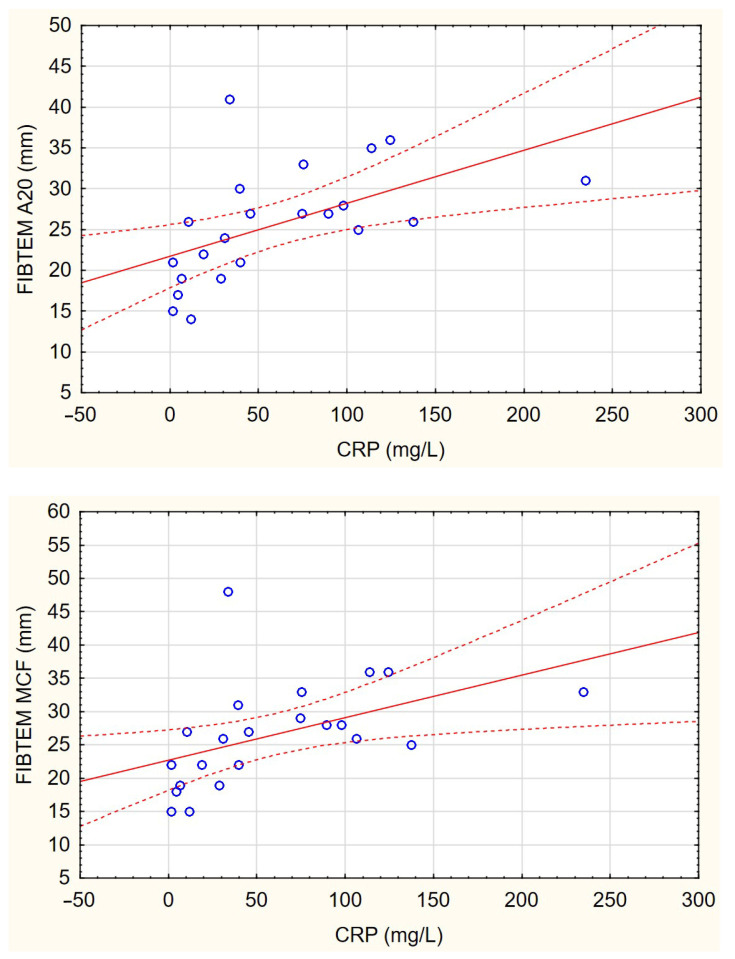
Correlations between FIBTEM test parameters and CRP level.

Most NATEM parameters, determining the dynamics of clot initiation (CT) and formation (CFT, CFR, and alpha) and the strength of the clot (MCE and MCF), correlated with the level of all inflammatory biomarkers (see Supplementary Table S1). In general, the higher the concentration of inflammatory biomarkers, the shorter the clot formation time and the greater the strength of the clot. The strongest correlations with inflammatory biomarkers were with the values of FIBTEM parameters (Figure 1). All analyzed FIBTEM parameters correlated with the levels of all inflammatory biomarkers. The values of parameters determining the phase of clot initiation and the dynamics of clot formation in the EXTEM test correlated only with some inflammatory biomarkers. The EXTEM parameters of clot strength mostly did not correlate with the levels of inflammatory biomarkers.

### 3.5. Multivariate Logistic Regression

ROTEM parameters and interleukin levels that differed significantly between the COVID-19 group and controls were included in the multivariate logistic regression model with death and ICU transfer as outcome variables. In addition, we included CT severity index, clinical category on admission, CRP level, and basic coagulation tests (PT, APTT, fibrinogen level, and D-Dimer) in the multivariate logistic regression model. In the univariate analysis, stepwise logistic regression recommended a model with seven parameters for predicting the risk of death: clinical category on admission, level of Il-8, NATEM MFC, NATEM MCE, FIBTEM MCF, FIBTEM MCE, and FIBTEM A20, and a model with four parameters for transfer to ICU/mechanical ventilation: CT severity score, FIBTEM MCF, FIBTEM MCE, and FIBTEM A20. Only the FIBTEM MCE value was significant in the model (Table 5), both as a predictor of death and transfer to ICU/mechanical ventilation. The model was validated via an χ^2^ test which confirmed the significance (*p* = 0.041 for death and *p* = 0.028 for ICU transfer). The R2 Nagelkerky coefficient was at a moderate level (0.38 for death and 0.32 for ICU transfer). The additional assessment with a Hosmer–Lemeshow GOF test (*p* = 0.1786 for death and *p* = 0.5446 for ICU transfer) confirmed the good fit of the model to the data.

**Figure 2:**
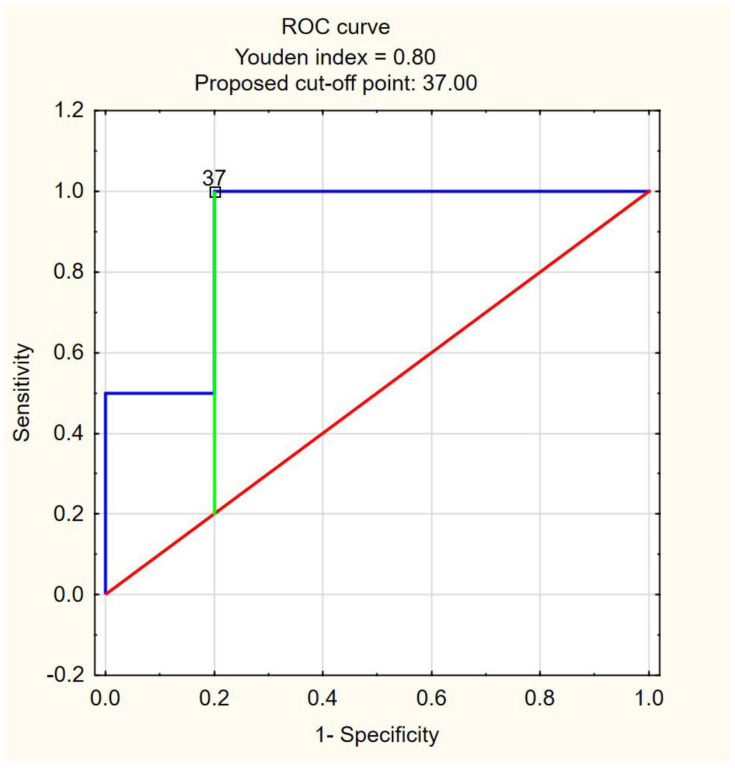
ROC curve for FIBTEM MCE as a predictor of death. The area under the curve (AUC) = 0.9, 95% confidence interval (95% CI): (0.7457; 1). The optimal cutoff point for FIBTEM MEC was 37, with death prediction for FIBTEM MCE ≥ 37. The sensitivity and specificity of the analysis were 100% and 80%, respectively. The accuracy of the test (ACC) was 80.8%.

**Figure 3:**
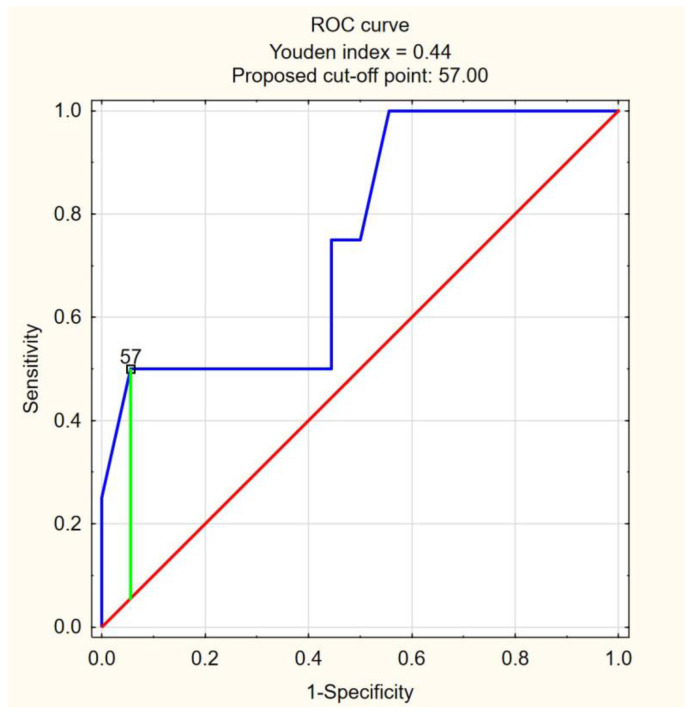
ROC curve for FIBTEM MCE as a predictor of transfer to ICU/mechanical ventilation. AUC = 0.75, 95% CI: (0.483; 1). The optimal cutoff point for FIBTEM MCE level was 57, with ICU transfer/mechanical ventilation prediction for FIBTEM MCE ≥57. The sensitivity of the analysis was 50%, and specificity was 94.4%. The ACC was 86.4%.

To ascertain the optimal cutoff point for FIBTEM MCE as a predictor for death or transfer to ICU/mechanical ventilation, ROC curves were prepared (Figure 2 and Figure 3). For FIBTEM MCE as a predictor of death, the analysis resulted in the area under the curve (AUC) = 0.9, 95% confidence interval (95% CI): [0.7457; 1]. The optimal cutoff point for FIBTEM MCE was 37 mm, with death prediction for FIBTEM MCE ≥ 37. The sensitivity and specificity of the analysis were 100% and 80%, respectively. The accuracy of the test (ACC) was 80.8%. For FIBTEM MCE as a predictor of transfer to ICU/mechanical ventilation, the analysis resulted in the AUC = 0.75, 95% CI: [0.4828; 1]. The optimal cutoff point for the FIBTEM MCE level was 57 mm, with ICU transfer/mechanical ventilation prediction for FIBTEM MCE ≥57 mm. The sensitivity of the analysis was 50%, and the specificity was 94.4%. The ACC was 86.4%.

## 4. Discussion

Although respiratory failure remains the leading cause of death from COVID-19, thromboembolic complications are a significant cause of morbidity and mortality, and their incidence increases with the severity of the disease. The use of low-molecular-weight heparins has significantly reduced the incidence of thrombotic complications in COVID-19 patients; however, their widespread use may have negative effects, including heparin-induced thrombocytopenia (HIT) and hemorrhagic events [7,16]. Taking into account the side effects of LMWH and the cost-effectiveness of treatment, the proper stratification of thromboembolic risk is crucial in patients with COVID-19. 

So far, many tests have been studied for their ability to predict thromboembolic complications in COVID-19 patients. Of the biomarkers of inflammation, the most evidential is the usefulness of CRP and D-dimer levels in predicting both the severity of COVID-19 and the risk of thrombotic complications. However, both biomarkers are non-specific and do not allow for the management of anticoagulation [17]. Markedly elevated levels of Il-6 correlate with both the severity of COVID-19 and poor prognosis [18]. Nevertheless, there is insufficient evidence for the usefulness of IL-6 in predicting thromboembolic risk. Basic coagulation tests (PT, APTT) are neither predictive of COVID-19 severity nor assessing thromboembolic risk. More sophisticated coagulation markers, including anti-Xa assays and thrombin generation are also not useful as predictors of prognosis or thromboembolic complications in COVID-19 patients.

Rotational thromboelastometry (ROTEM; Instrumentation Laboratories) is a viscoelastic method that allows the definition of a patient’s hemostatic profile [8]. In ROTEM, the tension between a pin suspended in a blood cup is recorded. This tension is displayed as clot strength (amplitude and maximum cloth firmness) in the time–amplitude plot. Independently, other parameters of the graph are recorded, which determine the dynamics of coagulation and lysis and the quality of the clot. ROTEM has some advantages over conventional laboratory coagulation assays. The test is performed on a whole blood sample and allows for a holistic overview of ex vivo coagulation. In practice, ROTEM^®^ assays with various coagulation activators are most often used, e.g., tissue factor (EXTEM^®^ test) or ellagic acid (INTEM^®^ test), to assess the extrinsic or intrinsic coagulation pathways, respectively. In turn, NATEM^®^ does not use additional activators. Due to its long reaction time, it is not routinely used clinically. It potentially allows for the detection of more subtle prothrombotic disorders compared to activated tests, as shown in previous studies in non-COVID patients [19]. 

In our study, ROTEM^®^ results indicated hypercoagulability in COVID-19 patients in all tests performed (NATEM^®^, EXTEM^®^, and FIBTEM^®^). Interestingly, the differences between the groups were more evident in the NATEM^®^ test compared to EXTEM^®^. The differences in NATEM^®^ were in all parameters analyzed, including the parameters of the coagulation initiation (CT and CFT), the dynamics of clot formation (CFR and alpha angle), and the clot quality (MCF and MCE). In the EXTEM^®^ test, the differences between the groups were less significant, and they were only in alpha and CFR parameters. Therefore, the NATEM test seems to be more sensitive for detecting hypercoagulability in COVID-19 patients compared to the EXTEM test. Overall, our ROTEM results are consistent with previous studies based on viscoelastic methods indicating a shorter initial clot generation time, more rapid clot formation, increased clot strength, and increased clot stability [20]. The novelty is that the use of non-activated ROTEM may be more useful in detecting prothrombotic disorders in COVID-19 patients compared to an activated test. Nevertheless, although the detection of hypercoagulability with the NATEM test looks promising and is much cheaper than activated tests, it requires further research.

FIBTEM^®^ is an EXTEM-based test that uses a platelet inhibitor (cytochalasin D) in addition to tissue factor activation. FIBTEM tests the quality of the platelet-free clot by detecting plasma coagulation disorders, including fibrin polymerization abnormalities and fibrinogen deficiency. The FIBTEM test results also indicated hypercoagulability in the COVID-19 patients, but unlike the NATEM and EXTEM results, the FIBTEM results were additionally correlated with the CT severity index. Moreover, among the ROTEM tests, the FIBTEM results correlated most strongly with inflammatory cytokine levels. Finally, elevated FIBTEM MCE was the strongest predictor of death and ICU transfer in the multivariate analysis. Our results are in line with preliminary data from other studies, indicating that fibrin cloth strength might be associated with prognosis [21]. Previous studies have also shown a relationship between fibrin clot strength in viscoelastic tests and the risk of thrombotic complications [20]. However, only one patient had thrombosis in our study, which makes it impossible to perform an appropriate analysis.

Comparing MCE in FIBTEM and EXTEM enables an assessment of the relative proportion of fibrin and platelets to cloth strength. We showed that the proportion of platelets in the clot in COVID-19 patients was comparable to the controls. Previous studies have indicated increased platelet activation in COVID-19 patients based on the level of soluble markers of platelet activation or flow cytometry [22]. However, studies based on aggregometry have not shown increased platelet aggregation in COVID-19 patients [23]. The relationship between increased platelet activation and thromboembolic complications in patients with COVID-19 has also not been confirmed. Overall, our study, based on ROTEM, indicates a greater contribution of plasma abnormalities than increased platelet activation to COVID-associated hypercoagulability.

We found no significant differences between the groups regarding fibrinolysis in ROTEM^®^. The ML parameter did not differ between the groups in the NATEM or EXTEM test. The relationship between thrombotic events and hypofibrinolysis detected by viscoelastic tests has been documented in COVID-19 patients hospitalized in ICUs [24]. Data on non-ICU patients are limited. Nevertheless, our results suggest that hypofibrinolysis is not common in patients who are not critically ill. Previous studies on classic markers of fibrinolysis, including PAI-1, tPA, TAFI, and thrombomodulin have suggested an association with COVID-19 severity [25,26]. However, the evidence for their association with thrombosis is inconclusive.

In our study, we also analyzed the levels of several cytokines, including interleukin-8 (IL-8), interleukin-1β (IL-1β), interleukin-6 (IL-6), interleukin-10 (IL-10), tumor necrosis factor (TNF), and interleukin 12p70 (IL-12p70). Although the levels of all of the above proteins were significantly higher in COVID-19 patients, none were better at predicting poor prognoses compared to routinely used CRP and IL-6. Moreover, the predictive value of inflammatory biomarkers was lower compared to the FIBTEM MCE of the ROTEM test.

Finally, in our study, we confirmed the usefulness of ROTEM for both the detection of hypercoagulability and the clinical course of COVID-19. Nevertheless, several aspects require further research. First, the persistence time of hypercoagulability detected in ROTEM after COVID-19 is not precisely defined. Previous studies on this topic showed the normalization of viscoelastic test parameters at 3 and 6 months in case series of ICU patients [21,27,28]. Nevertheless, to the best of our knowledge, this has not been studied in non-ICU patients. Second, the relationship between the hypercoagulability detected in ROTEM and the occurrence of thromboembolic incidents remains unclear, and the results of available studies provide conflicting results [12]. Both aspects could be examined in a prospective study with follow-up, evaluating both the ROTEM profile at specific intervals and the incidence of thromboembolic complications. Third, our study was conducted during the period of the predominant prevalence of the Delta variant of SARS-CoV2 in our country. Newer coronavirus variants appear to cause fewer thromboembolic incidents. Nevertheless, the analysis of ROTEM profiles in patients with COVID-19 caused by newer SARS-CoV2 variants requires further research. Finally, the usefulness of ROTEM to guide antithrombotic therapy requires further study.

Our study has several limitations. The first is the relatively small sample size. Nevertheless, the group was homogeneous in terms of severity and the treatment applied. Second, we did not routinely screen patients for thromboembolic complications. This means their prevalence may be underestimated. We were also unable to perform an analysis of coagulation disorders with thrombotic complications. Finally, ROTEM requires validation in COVID-19 patients, which currently limits its routine use.

## 5. Conclusions

In conclusion, in our study, we have demonstrated that certain parameters of ROTEM—the point-of-care viscoelastic method—can be useful for both detecting hypercoagulability and predicting the clinical course of non-ICU COVID-19 patients. Generally, our results suggest a greater contribution of plasma abnormalities than increased platelet activation to COVID-19-associated hypercoagulability. Of the ROTEM tests, the maximum elasticity of the fibrin clot (FIBTEM MCE) can be a useful predictor of COVID-19 severity. The value of inflammatory biomarkers—interleukin-8 (IL-8), interleukin-1β (IL-1β), interleukin-10 (IL-10), tumor necrosis factor (TNF), and interleukin 12p70 (IL-12p70)—in predicting poor outcomes was lower compared to the FIBTEM MCE and routinely used CRP and IL-6. In turn, ROTEM without additional clotting activators (NATEM test) may be a more valuable, cheaper, and simpler alternative for detecting hypercoagulability in COVID-19 patients compared to the tissue factor-activated EXTEM test. Our results also confirm previous reports suggesting that hypofibrinolysis is not common in patients who are not critically ill.

## Figures and Tables

**Table 1:** Clinical characteristics and results of basic laboratory tests of COVID-19 patients.

	COVID-19 Patients (*n* = 29)
Clinical category on admission to the hospital	
Asymptomatic	-
Hb saturation > 95%	6 (20.69%)
Hb saturation 91–95%	11 (37.93%)
Hb saturation < 90%	12 (41.38%)
BMI	29.95 ± 5.0
WBC (×10^3^/μL)	6.235 (5.1; 8.05)
Monocytes (×10^3^/μL)	0.59 ± 0.17
Lymphocytes (×10^3^/μL)	2.03 (1.53; 2.3)
PLT (×10^3^/μL)	232.91 ± 52.18
MPV (μm^3^)	10.4 (10; 11.4)
PDW	11.8 (10.9; 13.8)
CRP (mg/L)	39.75 (11.64; 113.85)
ALT (IU/L)	25 (18; 40)
Creatinine (mg/L)	0.92 (0.83; 1.33)
PT (s)	12.3 (12; 13.1)
INR	1.08 ± 0.08
APTT (s)	32.08 ± 3.23
D-dimer	696 (547; 1494)
Fibrinogen (mg/dL)	549.41 ± 185.84

Body mass index (BMI), white blood cells (WBC), platelet count (PLT), mean platelet volume (MPV), platelet distribution width (PDW), C-reactive protein (CRP), ALT, prothrombin time (PT), international normalized ratio (INR), activated partial thromboplastin time (APTT).

**Table 2:** Comparison of ROTEM parameters (NATEM, EXTEM, and FIBTEM test) between COVID-19 patients and the control group.

	Controls	COVID-19	*p*
NATEM^®^			
CT (s)	677 (618; 775)	584 (513.5; 652.5)	0.009
CFT (s)	202 (167; 237)	146 (127; 188.5)	0.0016
MCF (mm)	52 (50; 55)	59 (54.5; 62.5)	<0.0001
alpha (degrees)	54.85 ± 7.87	60.96 ± 9.75	0.0136
ML (%)	9.93 ± 5.20	11.14 ± 4.82	0.3721
CFR (degrees)	62 (57; 67)	68.5 (64.5; 73.5)	0.0141
MCE (dynes/cm^2^)	108 (98; 125)	142 (120.5; 167)	<0.0001
EXTEM^®^			
CT (s)	68.5 (64; 75)	72 (66; 82)	0.0587
CFT (s)	80.5 (71; 94)	73 (65; 83)	0.1101
MCF (mm)	63.23 ± 3.38	64.93 ± 5.5	0.1613
alpha (degrees)	74 (71; 75)	77 (76; 79)	0.0002
ML (%)	10 (9; 14)	10 (8; 13)	0.6140
CFR (degrees)	76 (74; 78)	78 (77; 80)	0.0113
MCE (dynes/cm^2^)	173.97 ± 25.48	191.79 ± 48.05	0.0837
FIBTEM^®^			
MCF (mm)	16 (14; 19)	26.5 (22; 31)	<0.0001
MCE (mm)	18 (16; 23)	36.5 (28; 45)	<0.0001
A10 (mm)	15.33 ± 3.25	23.77 ± 5.84	<0.0001
A20 (mm)	16.47 ± 3.40	25.64 ± 6.95	<0.0001

NATEM^®^ is a non-activated test where clotting is initiated by the contact of blood with the surface of the cuvette. EXTEM^®^ is a test for the analysis of blood coagulation after the activation of coagulation by tissue factor. FIBTEM^®^ is an EXTEM^®^-based assay with the addition of a platelet inhibitor (cytochalasin) used to analyze coagulation without platelets. CT—clotting time; CFT—clot formation time; CFR—clot formation rate; MCF—maximum clot firmness; ML—maximal lysis; A10, A20—amplitude in 10 min and in 20 min.

**Table 3:** Interleukin activity levels in the COVID-19 group and the control group.

	Controls	COVID-19	*p*
TNF (pg/mL)	1.32 (0.7; 3.21)	6 (2.8; 17.87)	<0.0001
IL-6 (pg/mL)	1.32 (0.76; 2.25)	45.65 (19.95; 199.76)	<0.0001
IL-1B (pg/mL)	0.06 (0.02; 0.08)	1.43 (0.76; 4.23)	<0.0001
IL-8 (pg/mL)	0.54 (0.07; 1.2)	16.68 (5.32; 44.67)	<0.0001
IL-10 (pg/mL)	0.25 (0.07; 0.65)	10.56 (3.23; 32.2)	<0.0001
IL-12p70 (pg/mL)	0.12 (0.043; 0.43)	2.76 (1.43; 7.98)	<0.0001

Interleukin-8 (IL-8), interleukin-1 (IL-1β), interleukin-6 (IL-6), interleukin-10 (IL-10), tumor necrosis factor (TNF), interleukin12p70 (IL-12p70).

**Table 4:** Correlations between CT severity score and FIBTEM^®^ parameters.

Correlated Parameters		R	*p*
CT severity index	FIBTEM MCF (mm)	0.520923	0.012924
	FIBTEM MCE (mm)	0.512226	0.014801
	FIBTEM A10 (mm)	0.483663	0.022573
	FIBTEM A20 (mm)	0.532274	0.010772

Spearman rank correlation of CT severity index and FIBTEM^®^ parameters. R—rank correlation coefficient. FIBTEM^®^ is an EXTEM^®^-based assay with the addition of a platelet inhibitor (cytochalasin) used to analyze coagulation without platelets. MCF—maximum clot firmness; MCE—maximum clot elasticity; A10, A20—amplitude in 10 min and in 20 min, respectively.

**Table 5:** Multivariate logistic regression model for death and transfer to ventilation in COVID-19 patients.

Characteristic	Coefficient	SE	*p*-Value	OR	95% CI for OR
Death					
FIBTEM MCE	2.81	0.05	0.094	1.09	0.99–1.20
Constant	5.24	2.67	0.022	0.01	0.01–0.42
ICU/need for mechanical					
FIBTEM MCE	2.88	0.05	0.0897	1.08	0.99–1.19
Constant	5.11	2.17	0.0248	0.01	0.01–0.52

SE—standard error; OR—odds ratio; 95% CI—95% confidence interval; FIBTEM^®^ is an EXTEM^®^-based assay with the addition of a platelet inhibitor (cytochalasin) used to analyze coagulation without platelets. MCE—maximum clot elasticity.

## Data Availability

The data that support the findings of this study are available from the corresponding author, [P.R.], upon reasonable request.

## Supplementary Materials

The following supporting information can be downloaded at: https://www.mdpi.com/article/10.3390/jcm12123919/s1, Supplementary Table S1. Correlations between ROTEM parameters and levels of inflammatory biomarkers.
