# Supplementary material for: Rotational Thromboelastometry (ROTEM®) in Relation to Inflammatory Biomarkers and Clinical Outcome in COVID-19 Patients
Source: J Clin Med. 2023 Jun 8;12(12):3919. doi: 10.3390/jcm12123919 (PMC10299160; doi:10.3390/jcm12123919)
Supplement: Supplementary file 1 [file jcm-12-03919-s001.zip › jcm-2380869-supplementary.pdf]

Supplementary table S1. Correlations between ROTEM parameters and levels of inflammatory biomarkers.

	R	p
NATEM CT (sec.) & CRP (mg/L)	-0.428356	0.022955
NATEM CT (sec.) & IL-10 (pg/mL)	-0.379208	0.004301
NATEM CT (sec.) & IL-6 (pg/mL)	-0.306414	0.022890
NATEM CT (sec.) & IL-1B (pg/mL)	-0.394239	0.002899
NATEM CT (sec.) & IL-8 (pg/mL)	-0.285828	0.034394
NATEM CFT (sec.) & IL-12p70 (pg/mL)	-0.310232	0.021162
NATEM CFT (sec.) & TNF (pg/mL)	-0.333303	0.012896
NATEM CFT (sec.) & IL-10 (pg/mL)	-0.473213	0.000264
NATEM CFT (sec.) & IL-1B (pg/mL)	-0.279765	0.038580
NATEM CFT (sec.) & IL-8 (pg/mL)	-0.287560	0.033270
NATEM MCF (mm) & CRP (mg/L)	0.560650	0.001914
**NATEM MCF (mm) & IL-12p70 (pg/mL)**	**0.522334**	**<0.0001**
NATEM MCF (mm) & TNF (pg/mL)	0.278446	0.039544
**NATEM MCF (mm) & IL-10 (pg/mL)**	**0.511767**	**<0.0001**
NATEM MCF (mm) & IL-6 (pg/mL)	0.402067	0.002343
NATEM MCF (mm) & IL-1B (pg/mL)	0.419856	0.001417
NATEM MCF (mm) & IL-8 (pg/mL)	0.401206	0.002400
NATEM alpha (degrees) & CRP (mg/L)	0.397368	0.036268
NATEM alpha (degrees) & TNF (pg/mL)	0.292197	0.030411
NATEM alpha (degrees) & IL-10 (pg/mL)	0.422021	0.001331
NATEM alpha (degrees) & IL-8 (pg/mL)	0.285085	0.034886
NATEM CFR (degrees) & IL-12p70 (pg/mL)	0.284031	0.035593
NATEM CFR (degrees) & IL-10 (pg/mL)	0.307002	0.022616
NATEM MCE (dynes/cm^2) & CRP (mg/L)	0.562860	0.001820
**NATEM MCE (dynes/cm^2) & IL-12p70 (pg/mL)**	**0.535362**	**<0.0001**
NATEM MCE (dynes/cm^2) & TNF (pg/mL)	0.271700	0.044790
**NATEM MCE (dynes/cm^2) & IL-10 (pg/mL)**	**0.514384**	**<0.0001**
NATEM MCE (dynes/cm^2) & IL-6 (pg/mL)	0.412780	0.001737
NATEM MCE (dynes/cm^2) & IL-1B (pg/mL)	0.432956	0.000962
NATEM MCE (dynes/cm^2) & IL-8 (pg/mL)	0.404101	0.002216
EXTEM CT (sec.) & CRP (mg/L)II	0.400069	0.042857
EXTEM CT (sec.) & IL-6 (pg/mL)	0.295946	0.022857
EXTEM CT (sec.) & IL-8 (pg/mL)	0.260863	0.045982
EXTEM CFT (sec.) & IL-8 (pg/mL)	-0.305788	0.018509
EXTEM MCF (mm) & CRP (mg/L)	0.423541	0.022052
EXTEM alpha (degrees) & CRP (mg/L)	0.400375	0.031382
EXTEM alpha (degrees) & IL-12p70 (pg/mL)	0.427308	0.000737
EXTEM alpha (degrees) & IL-10 (pg/mL)	0.393879	0.002025
EXTEM alpha (degrees) & IL-6 (pg/mL)	0.424929	0.000795
**EXTEM alpha (degrees) & IL-8 (pg/mL)**	**0.493428**	**<0.0001**
EXTEM CFR (degrees) & CRP (mg/L)	0.436724	0.017850

EXTEM CFR (degrees) & IL-12p70 (pg/mL)	0.299690	0.021111
EXTEM CFR (degrees) & IL-10 (pg/mL)	0.290536	0.025595
EXTEM CFR (degrees) & IL-6 (pg/mL)	0.360691	0.005010
EXTEM CFR (degrees) & IL-8 (pg/mL)	0.344645	0.007516
EXTEM MCE (dynes/cm^2) & CRP (mg/L)	0.430807	0.019646
FIBTEM MCF (mm) & CRP (mg/L)	0.681963	0.000473
FIBTEM MCF (mm) & IL-12p70 (pg/mL)	0.508551	0.000118
FIBTEM MCF (mm) & TNF (pg/mL)	0.345982	0.011992
FIBTEM MCF (mm) & IL-10 (pg/mL)	0.420477	0.001911
FIBTEM MCF (mm) & IL-6 (pg/mL)	0.598334	<0.0001
FIBTEM MCF (mm) & IL-1B (pg/mL)	0.400421	0.003267
FIBTEM MCF (mm) & IL-8 (pg/mL)	0.536531	<0.0001
FIBTEM MCE (mm) & CRP (mg/L)	0.682128	0.000471
FIBTEM MCE (mm) & IL-12p70 (pg/mL)	0.504063	0.000139
FIBTEM MCE (mm) & TNF (pg/mL)	0.336812	0.014625
FIBTEM MCE (mm) & IL-10 (pg/mL)	0.395377	0.003720
FIBTEM MCE (mm) & IL-6 (pg/mL)	0.574136	<0.0001
FIBTEM MCE (mm) & IL-1B (pg/mL)	0.412131	0.002399
FIBTEM MCE (mm) & IL-8 (pg/mL)	0.536136	<0.0001
FIBTEM A10 (mm) & CRP (mg/L)	0.741307	<0.0001
FIBTEM A10 (mm) & IL-12p70 (pg/mL)	0.506839	0.000126
FIBTEM A10 (mm) & TNF (pg/mL)	0.352115	0.010468
FIBTEM A10 (mm) & IL-10 (pg/mL)	0.455933	0.000682
FIBTEM A10 (mm) & IL-6 (pg/mL)	0.630412	<0.0001
FIBTEM A10 (mm) & IL-1B (pg/mL)	0.454460	0.000713
FIBTEM A10 (mm) & IL-8 (pg/mL)	0.615144	<0.0001
FIBTEM A20 (mm) & CRP (mg/L)	0.713438	0.000193
FIBTEM A20 (mm) & IL-12p70 (pg/mL)	0.507331	0.000124
FIBTEM A20 (mm) & TNF (pg/mL)	0.345396	0.012147
FIBTEM A20 (mm) & IL-10 (pg/mL)	0.457432	0.000651
**FIBTEM A20 (mm) & IL-6 (pg/mL)**	**0.642745**	**<0.0001**
FIBTEM A20 (mm) & IL-1B (pg/mL)	0.467784	0.000471
**FIBTEM A20 (mm) & IL-8 (pg/mL)**	**0.625651**	**<0.0001**

Spearman rank correlation of ROTEM parameters and inflammatory biomarkers. R - rank correlation coefficient. C-reactive protein (CRP), Interleukin-8 (IL-8), interleukin-1 (IL-1β), interleukin-6 (IL-6), interleukin-10 (IL-10), tumor necrosis factor (TNF), interleukin12p70 (IL-12p70). NATEM® is a non-activated test where clotting is initiated by contact of blood with the surface of the cuvette. EXTEM® is a test for the analysis of blood coagulation after activation of coagulation by tissue factor. FIBTEM® is an EXTEM® - based assay with the addition of a platelet inhibitor (cytochalasin) used to analyze coagulation without platelets. CT - clotting time, CFT - Clot Formation Time, CFR - Clot Formation Rate, MCF - Maximum Clot Firmness, ML - maximal lysis, A10, A20 - Amplitude in 10 minutes and in 20 minutes.
